# Supplementary material for: A digital dashboard for reporting mental, neurological and substance use disorders in Nairobi, Kenya: Implementing an open source data technology for improving data capture
Source: PLOS Digit Health. 2024 Nov 1;3(11):e0000646. doi: 10.1371/journal.pdig.0000646 (PMC11530017; doi:10.1371/journal.pdig.0000646)
Supplement: S1 Appendix — (DOCX) [file pdig.0000646.s001.docx]

**S1 Appendix: Screenshots of different sections of the dashboard**


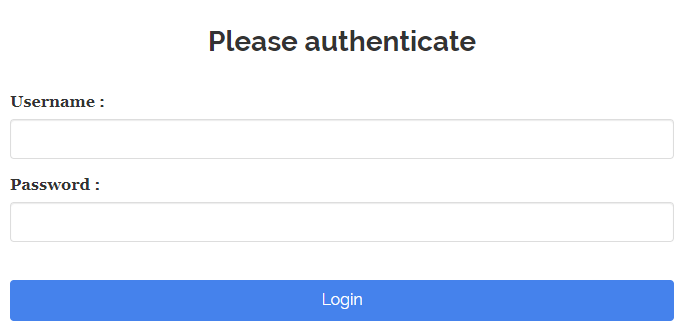

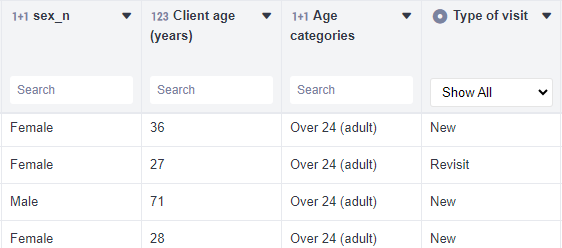


**1**

**2**


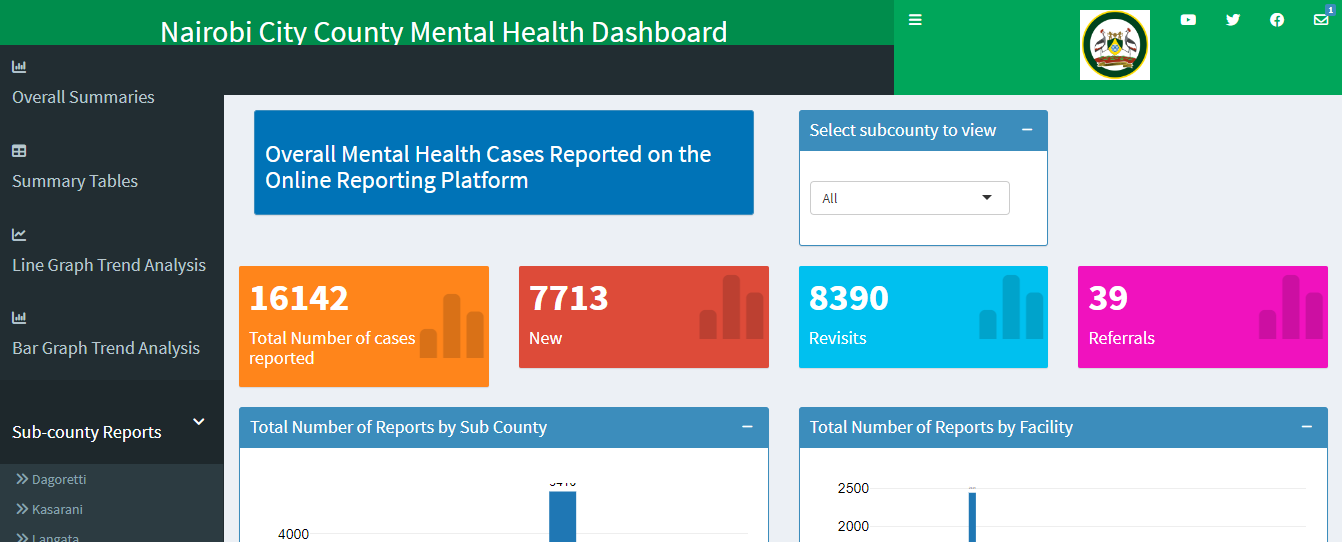


**3**

**5**

**4**


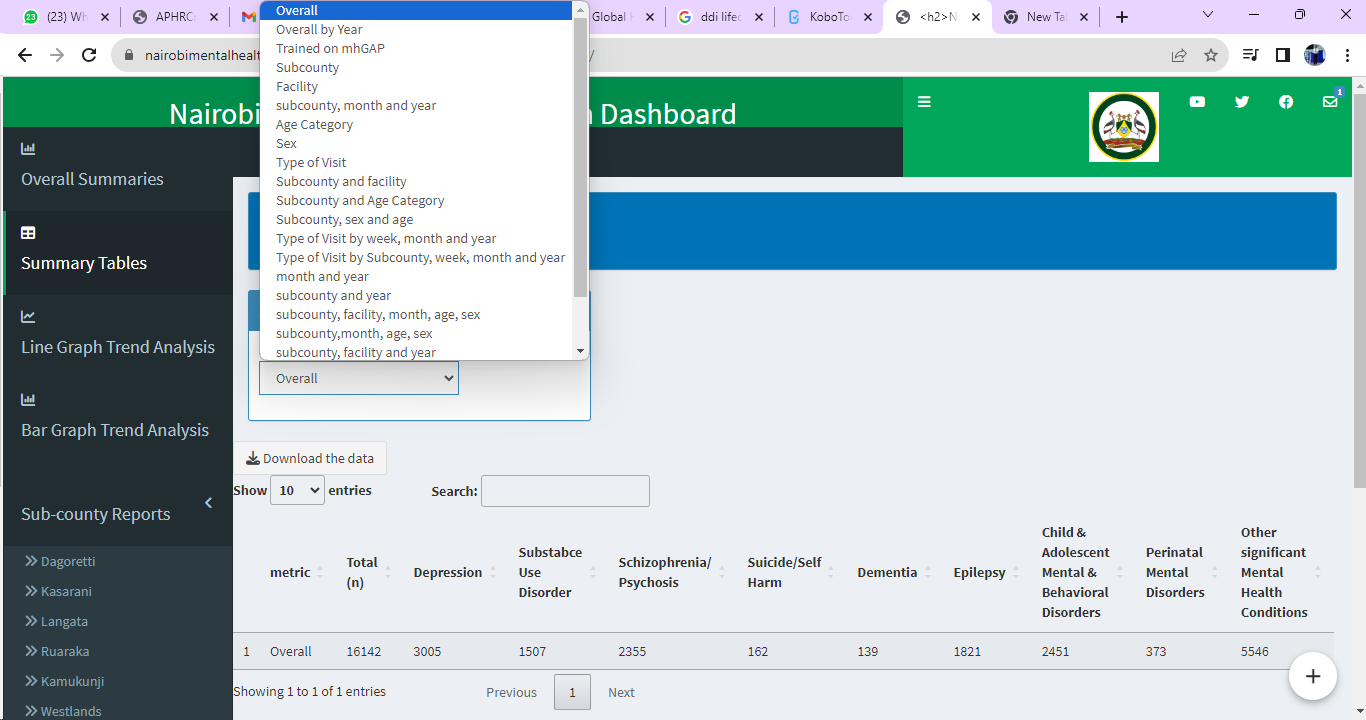

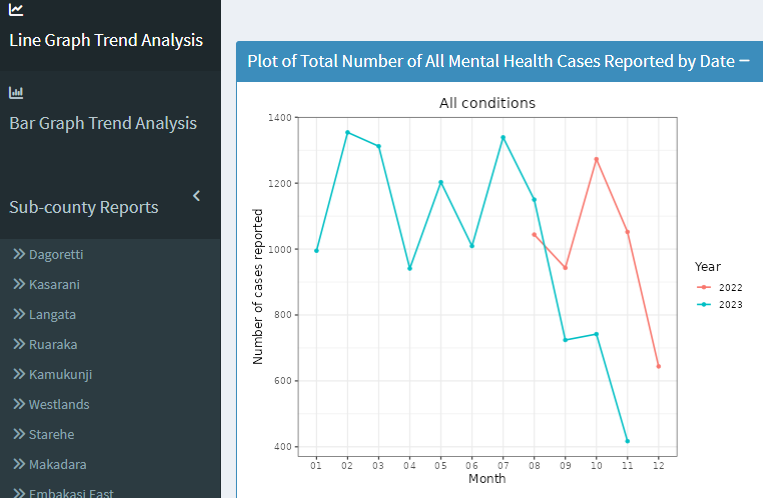


Fig A: Screenshots of the digital reporting system and the dashboard
