# Supplementary material for: A digital dashboard for reporting mental, neurological and substance use disorders in Nairobi, Kenya: Implementing an open source data technology for improving data capture
Source: PLOS Digit Health. 2024 Nov 1;3(11):e0000646. doi: 10.1371/journal.pdig.0000646 (PMC11530017; doi:10.1371/journal.pdig.0000646)
Supplement: S2 Appendix — (DOCX) [file pdig.0000646.s002.docx]

**S2 Appendix: Integration of R and Kobo tool box**

**Installation of packages**

## install.packages(“remotes”)

##remotes::install_github(“dickoa/robotoolbox”)

library(robotoolbox)

**Getting the API content and setting up the session**

[kobo_token](https://dickoa.gitlab.io/robotoolbox/reference/kobo_token.html)(username = "xxxxxxxxx",

password = "xxxxxxxxx",

url = "https://kobo.xxxxxxxxx.org")

[kobo_setup](https://dickoa.gitlab.io/robotoolbox/reference/kobo_setup.html)(url = "https://kobo.xxxxxxxxx.org",

token = "xxxxxxxxxxxxxxxxxxxxxxxxxx")

[kobo_settings](https://dickoa.gitlab.io/robotoolbox/reference/kobo_settings.html)() ## Check settings to confirm if the token has been correctly extracted and linked

With the settings done, listing all assets associated with that account is possible. That is, the data collection forms uploaded to that account and their properties, including the form IDs, type, owner of the form, date created and date when the form was modified.

list_projs <- kobo_asset_list()

> glimpse(list_projs)

Rows: 1

Columns: 7

$ uid <chr> " xxxxxxxxx "

$ name <chr> " xxxxxxxxx "

$ asset_type <chr> "survey"

$ owner_username <chr> "xxxxxxxxx"

$ date_created <dttm> 2022-05-12 20:44:39

$ date_modified <dttm> 2022-09-01 14:29:04

$ submissions <int> xxxxxxxxx

The list of assets is a tibble which allows you to to select the form uuid that uniquely identify the project you want to open. The function kobo_asset() can then be used to get the asset from the uuid and the function [kobo_data](https://dickoa.gitlab.io/robotoolbox/reference/kobo_data.html)()

> uid <- list_projs$uid[1]

> ncc_report_v4 <- kobo_asset(uid)

> ncc_report_v4

<robotoolbox asset> “xxxxxxxxx”

Asset name: NCC Mental Health Reporting Tool

Asset type: survey

Asset owner: xxxxxxxxx

Created: 2022-05-12 20:44:39

Last modified: 2022-09-01 14:29:04

Submissions: 5296

uid <- list_projs$uid[1]

data_asset <- kobo_asset(uid)

Now with the selected asset, we can extract the submissions using the kobo_submissions() function or the [kobo_data](https://dickoa.gitlab.io/robotoolbox/reference/kobo_data.html)() function. Once the data has been extracted from the database, the usual data management procedures follow depending on the specific data management and analysis needed.

#df <- [kobo_submissions](https://dickoa.gitlab.io/robotoolbox/reference/kobo_data.html)(asset) ## or df <- kobo_data(asset)

#glimpse(df)

dataset <- [kobo_data](https://dickoa.gitlab.io/robotoolbox/reference/kobo_data.html)(data_asset) ##Can also use kobo_submissions() function

> glimpse(dataset)

Rows: 17185

Columns: 45

**The Shiny dashboard Syntax**

library(shiny)

library(shinydashboard)

ui <- dashboardPage(

dashboardHeader(),

dashboardSidebar(),

dashboardBody()

)

Server <- function(input, output) { }

shinyApp (ui, server)

**Hosting of the dashboard**

Shiny applications can be hosted in the shinyapp.io platform [19], an easy and self-service platform that enables users and developers to share their R Shiny applications on the web. The free version of shinyapps.io allows only a maximum of 5 applications and 25 active hours. If more than 5 applications are desired, users may need to subscribe at a fee based on the number of potentially hosted applications. The free version of shinyapps.io is useful for applications that are not busy or as a test environment for the dashboard during development.

An alternative approach is to host the dashboard on a local server, but the shiny server and RStudio servers have to be installed in the local server running on Linux. The dashboard is then accessed through a link generated when it is uploaded to local hosting platform. The requirements for the dashboard to be hosted on a local server include having a Linux server virtual environment running on 6 CPUs, 6 GB Memory and 500 GB Storage space. The software requirements are *R and RStudio, Shiny Server,* *unrar*, *httpd, mariadb-server, mariadb, php, php-mysql, mod_ssl* and *phpmyadmin*, and an expert with Linux environment management and implementation skills. There is no cost subscription for the server hosted locally to run. Suppose the above requirements are not met to host the dashboard locally. In that case, shinyapps.io is a good option for the free version (no more than 5 applications are uploaded) or subscribed version, depending on the number of applications to be hosted. Currently, the dashboard is hosted on a local server and a back-up system on *shinyapps.io*.
