# Supplementary material for: A digital dashboard for reporting mental, neurological and substance use disorders in Nairobi, Kenya: Implementing an open source data technology for improving data capture
Source: PLOS Digit Health. 2024 Nov 1;3(11):e0000646. doi: 10.1371/journal.pdig.0000646 (PMC11530017; doi:10.1371/journal.pdig.0000646)
Supplement: Attachment — Submitted filename: Response to Reviewers_PLOSDH_18Jun2024.docx [file pdig.0000646.s005.docx]

**Response to reviewer comments**

Reviewer #1: Summary:
goal is to use shiny/kobo dashboard to view and submit health care data about MNS disorders in real time. the system shows better real-time performance, as it was not aggregated and reported retrospectively in month increments (real time is more timely, accurate, and comprehensive). This work is aimed to build upon a previous manual tool, and to evaluate improvements using this new system

positives:
- i think this is a very relevant work for this venue.
- this work shows the importance of real-time reporting, data visualization for public health, and how digitization can improve growing health systems
- the authors did well at "setting the scene" with the previous monthly reporting method, describing the challenges with routing data through "focal persons"
-authors did well to describe how the tool was made and why they selected the technologies they did (especially for shiny)
- I appreciated that the dashboard was described verbally in layout (lines 192-198) this improves accessibility/reproducibility
- Pilot testing was well described with lessons learned clearly indicated.
-flowed well and was easy to follow step by step
- table 3 is great and clear to see differences in the systems.
- i appreciated the qualitative reviews from focal persons, i would be curious to hear if there were any focal persons who did not like the digital system more than the manual system.

Thank you for this feedback. It is much appreciated.

changes requested:
-would recommend rework of keywords to be more general

Response: This has been noted and updated

- are there age limits for reporting? is this adults and children? More information on the populations/facilities would be helpful.
Response: Thank you for this question. There were no age limits in reporting. We have provided additional information on the study populations.

- I was surprised to see that many facilities still did not report, even with this new system. It also looked like less and less facilities were reporting as time progressed... can the authors reference this trend or describe why we are still seeing low compliance rates?
Response: This is a great observation. We have provided a context has been provided in the discussion.

-table 1 format is strange and a bit difficult to read without a column divider between the two primary sections. please reformat so we can clearly compare the manual versus digital system.
Response: This is noted. We have added columns to separate the two main sections.

-spelling error in media on line 269
Response: Thank you for catching this. This is noted – we have corrected it.

- i am unclear on what is different in the data being shown in table 1 and table 2 - please make it clearer what we are comparing across these two tables and what makes them different. I can see the data is different but it is not abundantly clear why.
Response: The two tables were presented separately because the first table shows data during the pilot phase and the second table presents data during the implementation phase.

- it would be helpful to understand the financial burden of implementing this new system compared to the previous system (how expensive would this be for another group to do in terms of money, personal, resources, etc). this was touched on a bit in the discussion but could be dived into more.
Response: This is a great point. Yes, this will be a useful information for scale up. While we did not specifically measure how much it would cost to implement the system, we have provided some context to it and general recommendations on what someone interested can do to implement.

- i would be curious to hear next steps and what other data is planned to be incorporated next and why
Response: Thank you for the comment. Yes, the system is still being improved as we go along, and we have started working on incorporating geospatial data into the system to see distribution of cases geospatially.

- i feel it is a significant downfall of the work that they don't have a screenshot or visualization of this dashboard or of some of the data collected. please add this in your next revision to help readers understand the dashboard beyond just the verbal description provided within text.
Response: Thank you for this comment. The screenshots are actually there, but are put in the appendix and reference to them are included in the methods section. We have edited the text in the main text to say the screenshots are in the appendix.

- can the authors provide any additional numerical/quantitative analyses or statistical tests to show/demonstrate, beyond just summary counts, the significance in additional data being collected with this new system.
Response: This has been added. P-values from two-sample t-tests have been added to show statistical significance for the comparison of data between the manual and the digital system.

Reviewer #2: This paper describes a digital tool for reporting mental health services provided in Nairobi, Kenya. Overall, its well-structured, well-written, and relevant to PLOS Digital Health. I have a few minor issues with the paper that the authors should be able to address easily.

Thank you so much for this feedback, this is much appreciated.

1. Introduction line 86: Why the sudden focus on perinatal mental health disorders specifically? Is the digital health tool being developed specific to PMH disorders (if so, were there any specific considerations made for this specific subset of mental health disorders), or is that the focus of the existing reporting tool? Of note, as far as I can tell, the acronym PMH is not used anywhere else in the paper outside of this paragraph. The authors should motivate the introduction of this concept.

Response: This is a great observation, thank you. We have added the context to its addition. It is mainly because mhGAP-IG, which is the guide used by Nairobi City County for its mental health program, does not have perinatal mental health. This was particularly added for Nairobi based on the justification provided. So, we have edited the paragraph and added something for clarity.

2. Introduction line 96: This sentence is the motivation for this entire work and needs to be backed up in some way. Are there other studies, national/international surveys, government reports, etc. that show that the existing system suffers from “late submissions, inaccurate aggregations, incomplete data and difficulties in analysing the data for timely use…”?

Response: Thank you. This is true. Another motivation of this work is that there is need for developing systems to generate data for mental health that are timely and reliable for timely decisions. In Kenya, the DHIS2 system used nationally reports mental health under three conditions (epilepsy, mental health, substance use). So, the team in Nairobi County decided to develop a tool to collect data which was reported at the end of each month after the subcounty teams aggregate the numbers seen on that month. It was manual, submitted on email to the county mental health coordinator and was prone to the errors as highlighted. So, there was no published survey, but the idea to digitize the work came from a need. And we saw it well to document our experience and share lessons with others through this paper. We have added a sentence to beef up the motivation in the main text.

3. Materials and Methods line 132: The authors note that they “digitized the data collection tool” here. Is this the same as the “reporting tool” mentioned and described on line 84? If so, the authors should pick one of the terms and use that terminology throughout the paper. If not, it would be helpful to readers to (at least briefly) describe here what that tool entails so they have some context as to what exactly is being digitized.

Response: Thank you for this observation and suggestion. Yes, the two terms have been used interchangeably. We have updated it to use reporting tool consistently.

4. Materials and Methods line 195: The authors mention that the spatial distribution page is still being developed, what is the spatial distribution page? It isn’t mentioned anywhere else and I’m not clear as to what functionality it is supposed to provide. It doesn’t seem to be providing any functionality key to the evaluation of the system, so perhaps this should be moved to the discussion where the authors describe functionality to be added?

Response: I agree, it is a bit misplaced. This has been removed from here and added to the discussion section.

5. Tables 1 and 2: These tables are a bit difficult to parse, and it took me some time to understand what I was supposed to be taking away from the table. I think adding some additional border lines and bolding would go a long way towards guiding readers towards what columns to compare and key takeaways. I also don’t understand first symbol in the notes in these tables. The authors state that this symbol indicates the total number reported in the period for the digital system, does that mean the referrals and total are not the total number reported in the period? For example, my understanding is that the first data row of Table 1 indicates that there were 499 new cases, 540 repeat visits, and 5 referrals during the month of August; does the 5 mean something different?

Response: This is a great observation. Apologies, there was an oversight – we forgot to add the symbol on referrals as well. We have done this and added something on the notes to make it clearer. We have also added boarders to help with comparisons, added statistical tests and also re-arranged the columns a little to make it clearer. Thank you for this.

6. Somewhere in the paper (perhaps in the introduction or discussion?) it would be beneficial to add a sentence or two discussing why having data on new cases, repeat visits, and referrals rather than just the total is useful/valuable to have.

Response: Great! This is noted and has been added in the introduction section.

7. Discussion: In thinking of what a reader would take away from this work, I would like to see an additional paragraph/section in the discussion section that clearly lays out the design considerations that were learned by the authors from this work that could be used by readers to develop their own similar system. If design considerations were specific to this system, I’d like some thought/insight into how they might generalize or be used for other systems.

Response: Thank you for this suggestion, which is an important addition to ensure the reader is able to replicate it if interested. I have added a paragraph in the discussion section.

Grammar/Minor Things:
Line 82: Missing period, should be “disorders. Currently, there are…”

Response: Thank you. This has been added.

Line 92: Missing a subject, who reported and aggregated data monthly?

Response: Thank you. Yes, the sentence was incomplete. Apologies. This has been corrected.

Line 288: “in the digital system and a quarter (23%).” Should this end with “and a quarter (23%) in the manual system.”?

Response: Thank you. Yes, correct. This has been added.

Table 3: Why is “Month” in the top left cell of this table?

Response: That was misplaced, thank you for catching this. This has been added.

--------------------

6. PLOS authors have the option to publish the peer review history of their article ([what does this mean?](https://journals.plos.org/digitalhealth/s/editorial-and-peer-review-process#loc-peer-review-history)). If published, this will include your full peer review and any attached files.
Response: This is okay.

**Do you want your identity to be public for this peer review?** If you choose “no”, your identity will remain anonymous but your review may still be made public.


For information about this choice, including consent withdrawal, please see our [Privacy Policy](https://www.plos.org/privacy-policy).


Reviewer #1: No

Reviewer #2: Yes: Josh Cherian

--------------------


[NOTE: If reviewer comments were submitted as an attachment file, they will be attached to this email and accessible via the submission site. Please log into your account, locate the manuscript record, and check for the action link "View Attachments". If this link does not appear, there are no attachment files.]

While revising your submission, please upload your figure files to the Preflight Analysis and Conversion Engine (PACE) digital diagnostic tool, https://pacev2.apexcovantage.com/. PACE helps ensure that figures meet PLOS requirements. To use PACE, you must first register as a user. Registration is free. Then, login and navigate to the UPLOAD tab, where you will find detailed instructions on how to use the tool. If you encounter any issues or have any questions when using PACE, please email PLOS at figures@plos.org. Please note that Supporting Information files do not need this step.

__________________________________________________
In compliance with data protection regulations, you may request that we remove your personal registration details at any time. (Use the following URL: https://www.editorialmanager.com/pdig/login.asp?a=r). Please contact the publication office if you have any questions.
